# Analysis of Spatial and Temporal Changes of AQI in Wuhan City under the Urban Blockade of COVID-19 Pandemic

**DOI:** 10.3390/ijerph19148350

**Published:** 2022-07-08

**Authors:** Nai Yang, Xin Sun, Yi Chao

**Affiliations:** School of Geography and Information Engineering, China University of Geosciences, Wuhan 430078, China; yangnai@cug.edu.cn (N.Y.); sun.xin@cug.edu.cn (X.S.)

**Keywords:** Coronavirus Disease 2019, air quality, lag effect, spatial-temporal differentiation, environmental impact

## Abstract

Wuhan was the primary city in the world to adopt lockdown measures during the Coronavirus Disease 2019 pandemic. The influence of the abrupt halt of human activities on the air quality of Wuhan is a subject of considerable attention. This study is based on air quality data from 21 monitoring stations in Wuhan from 2016 to 2020. The lag effect and seasonal factors of AQI were taken into account to analyze the changes in air quality in Wuhan under the influence of the pandemic blockade. The results showed the following during the city closure: (1) A lagging effect is observed in air quality changes, with the change point occurring on the 14th day after the city closure; (2) the air quality index is substantially decreased, demonstrating a reduction in spatial differences; (3) NO_2_, PM_10_, and PM_2.5_ significantly decreased whilst O_3_ increased, and SO_2_ and CO did not change significantly; (4) except for the insignificant changes in spatial differences of PM_10_, all pollutants demonstrated a changing pattern of decreasing geographical differences. This paper provides a reference for studying the influence of human activities on the natural environment.

## 1. Introduction

Over the past two years, Coronavirus Disease 2019 (COVID-19) pandemic has seen an increasing number of confirmed cases and deaths, resulting in incalculable loss of life and property. According to data from the World Health Organization on 29 June 2022 at 00:30 a.m. BST, the number of confirmed cases reached 542,188,789 and the number of confirmed deaths reached 6,329,275 worldwide [1]. Many countries or regions have adopted strict pandemic prevention measures to prevent the further spread of COVID-19; amongst which, minimizing the movement of people has become an effective means of prevention. Strict closure measures have serious negative impacts on social and economic aspects worldwide [2]. In the early stages of the pandemic, China experienced negative GDP growth of −6.8% year-on-year in the first quarter of 2020 [3]. However, the blockade has a positive effect on air quality improvement [4,5,6]. Wuhan is in central China, with a population of approximately 12.33 million [7] in an area of 8569.15 km^2^ [8]. In China, COVID-19 first broke out in Wuhan. The city adopted the most stringent city-wide lockdown strategy during the initial phase of the outbreak: public transportation operations were halted from 10 a.m. on 23 January 2020 [9]; streets, neighborhoods and villages were blocked; travel restrictions were imposed. These operations were lifted at 00 a.m. on 8 April, ending the city closure that lasted 76 days. All medical and household supplies were jointly distributed in Wuhan during this period [10], all residents were isolated at home and human activities in the entire city came to a near standstill, demonstrating considerable sacrifices to prevent the spread of the virus. Wuhan, the first city in the world to adopt a city closure strategy, offers an opportunity to study the impact of human activities on the atmospheric environment.

In addition to Wuhan, many countries and regions also adopted city closure measures during the COVID-19 outbreak to interrupt the spread of the virus. For example, Italy, Germany, France, New Zealand, India, and other countries either completely banned the movement of people or restricted activities in some areas at different times. Several scholars have studied the changes in air quality in these cities during the blockade period, and most of these studies have shown that urban closure has a positive effect on air quality improvement. Bassani, Filonchyk, Shafeeque and Arshad et al. used satellite remote sensing imagery to investigate changes in air pollution during the blockade period in Rome (March–April 2020), Poland (March–April 2020) and the Indo-Pak region (March–June 2020) respectively. The studies found that (1) the closure measures significantly reduced NO_2_ emissions from road traffic in Rome and surrounding areas and that the decrease was higher in urban than in rural areas [4]; (2) the closure measures significantly improved air quality in Poland [5]; (3) a significant decrease in air pollution could be observed across the Indo-Pak region during the blockade period [2]. They concluded that strict closure measures contributed to the improvement of global environmental conditions [6]. Malhotra and El-Sayed et al. studied the impact of the blockade on air quality in Delhi hot spots in India (March–April 2020) and six major cities in Florida (February–April 2020) through statistical analysis. They observed that reductions in pollutants PM_2.5_, PM_10_ and NO_2_ in Delhi, India, were significantly associated with the city closure measures [11], whilst NO_2_ and CO levels in Florida were reduced during the blockade [12]. Cui, Cao and Feng et al. studied the air quality in Dongying City, Shijiazhuang City and Xi’an City of China during and before and after the city closure. They found that (1) urban blockade had an improving effect on air quality in Dongying during the pandemic [13]; (2) the blockade reduced the emission of pollution sources to a certain extent and the pollutant concentrations of PM_2.5_, PM_10_, NO_2_, SO_2_ and CO all decreased in Shijiazhuang [14]; (3) NO_2_ levels in Xi’an substantially decreased during the city closure [15]. Tao et al. used a TVP-VAR model to analyze the dynamic relationship between pandemic, economy and air quality in Beijing from January to August 2020, showing the positive impact of the urban blockade on air management. However, this impact will gradually diminish as the blockade ends [16].

However, a few research results prove that urban blockade does not possess a positive effect on air pollution improvement. Donzelli et al. evaluated the impact of pollutant emission reductions on air quality in three cities during the Italian city closure period (March–June 2020) [17]. They concluded that evidence of a direct relationship between the implementation of closure measures and the reduction of particulate matter in urban centers (except in high traffic areas) is absent. However, they agreed that overall source control measures should be implemented to improve urban ambient air quality [17]. In addition, studies by Cao and Feng et al. have found some negative effects of city closure, reflected in the increase in O_3_ pollution [14,15].

The city of Wuhan is closed for two seasons, namely winter and spring, and the length of the blockade and the strict measures are unparalleled in many cities. Accordingly, systematically examining the effects of closure measures on air quality in Wuhan is particularly necessary. Seasonal factors are also an important factor affecting AQI [18,19]. In addition, air quality change due to the city closure is a slow process with a lag effect, which is not addressed in the existing relevant studies. The above lag effect and seasonal factors will be considered in this paper, and five years of air quality data of Wuhan city from 2016 to 2020 are selected to analyze the air quality lag effect and spatial and temporal divergence characteristics during the blockade of Wuhan city for quantifying the extent of human activities on urban air quality.

## 2. Materials and Methods

### 2.1. Materials Sources and Processing

**Figure 1:**
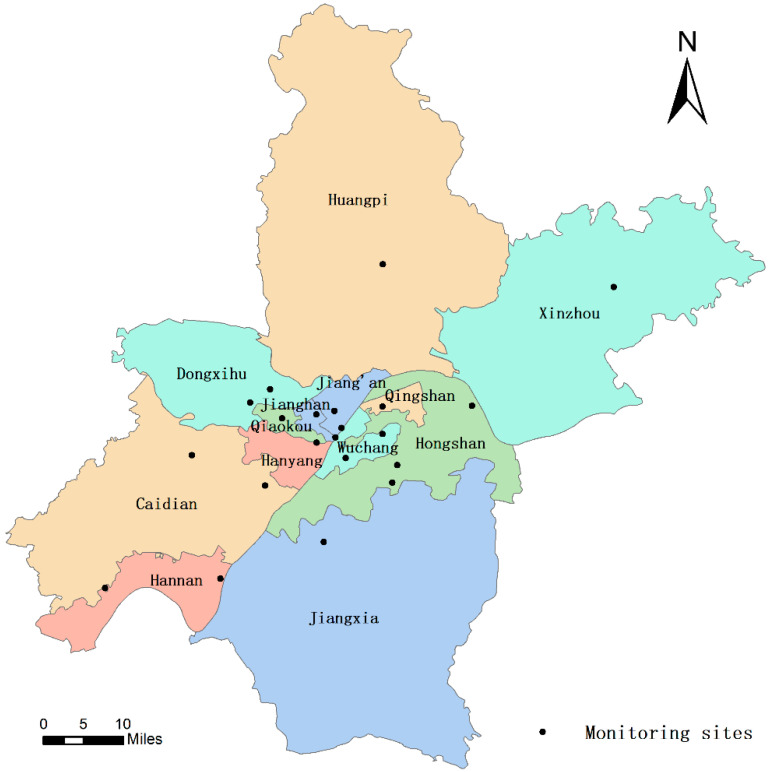
Distribution map of Wuhan City environmental monitoring sites.

The air quality data used in this paper were obtained from the official website of the Wuhan Ecological Environment Bureau, and the distribution of 21 environmental monitoring points in Wuhan is shown in Figure 1. This paper presents the atmospheric environmental quality data for 2020 during the closure of Wuhan and the same period of four years from 2016 to 2019 (i.e., the period from 23 January to 7 April, sulphur dioxide (SO_2_), nitrogen dioxide (NO_2_), inhalable particulate matter (PM_10_), carbon monoxide (CO), ozone (O_3_) and fine particulate matter (PM_2.5_)) to conduct a comparative study. The air quality index (AQI) value is the maximum value of the six pollutants, and the calculation formula and classification are referred to the official website of the Wuhan Ecological Environment Bureau as follows:

(1) $$\mathrm{AQI}=max\left\{V_{{\mathrm{SO}}_{2}},V_{{\mathrm{NO}}_{2}},V_{{\mathrm{PM}}_{10}},V_{\mathrm{CO}},V_{O_{3}},V_{{\mathrm{PM}}_{2.5}}\right\}$$

where *V* refers to the value of pollutant. Pollutants concentration is the mass of pollutants contained in each cubic meter. AQI is divided into the following six levels to facilitate the statistics of air quality excellent rate: 0 ≤ AQI ≤ 50 for excellent, 50 < AQI ≤ 100 for good, 100 < AQI ≤ 150 for light pollution, 150 < AQI ≤ 200 for moderate pollution, 200 < AQI ≤ 300 for heavy pollution and AQI > 300 for severe pollution. The data were pre-processed as follows. The missing data were calculated by the arithmetical series formula using the data that can be collected directly before and after this time period for which missing data exists; for the case of AQI values but with some zero pollution values, the pollution values were zero by default and were not processed. Finally, the AQI values were recalculated after supplementing all of the data. Details of the data are shown in Table 1.

### 2.2. Methods

#### 2.2.1. Pettitt Mutation Test

There is a time lag in the effect of a sudden cessation of human activity on changes in air quality. This delay can be reflected by data anomalies, i.e., change points. The change point is a point in the time series where the AQI changes suddenly and the probability distribution around the point no longer satisfies consistency [20]. It is mainly caused by sudden changes in external factors, such as the effects of climate change and human activities, and can reflect some kind of qualitative change in things [20]. Pettitt mutation test is a non-parametric mutation point detection method. This method is used in the current study to identify the mutation points of AQI during the city closure and analyze the lag effect of city closure on AQI. Firstly, for the time series data containing *n* samples, that is, *x_i_*, *i* = 1, 2, 3, …, *n*, the test statistic variable *U_t_* is defined and calculated as follows [21].

(2) $$U_{t,n}=U_{t-1,n}+V_{t,n},\;1\leq t\;<\;n$$

(3) $$V_{t,n}=\overset{n}{\underset{j=1}{\sum }}sgn\left(x_{t}-x_{j}\right)$$

Amongst them,

(4) $$\mathrm{sgn}\left(\theta \right)=\left\{\begin{array}{c} 1,\;\;\theta >0\\ 0,\;\;\theta =0\\ -1,\;\;\theta <0 \end{array}\right.$$

where the value of *x_i_* is the AQI value, *n* has two scenarios in different years, with values of 75 or 76 days.

And then, taking the maximum value of the absolute value in *U_t_*, *K_t_*, the point is the most significant possible mutation point. The statistic *P* corresponding to *K_t_* is calculated, corresponding to the significance probability calculation formula is:

(5) $$P=2e^{\frac{-6K_{t}^{2}}{n^{3}+n^{2}}}$$

If *P* is less than a given significance level (e.g., α = 0.05), then *P* indicates the presence of a statistically significant mutation point.

#### 2.2.2. Spatial Interpolation Analysis

Spatial interpolation is often used to convert measurements from discrete points to continuous data surfaces for comparison with the distribution patterns of other spatial phenomena [22]. The Kriging interpolation method considers the variation of spatial attributes based on spatial location relations and autocorrelation and uses the structure of variance functions of sample data points in regionalized variables to produce unbiased and optimal estimates of the values of the sample points to be estimated [23]. This method also has a good smoothing effect and can reduce the impact of extreme outliers on the overall distribution pattern of the sample [24]. The weekly averages of the processed AQI and various air pollutant values at 21 monitoring locations will be used in this study as interpolation attributes to obtain a trend surface result map through ordinary Kriging interpolation. The spatial and temporal changes in atmospheric conditions in Wuhan City from 2016 to 2020 are also analyzed, and the formula is respectively presented as follows [25]:

(6) $$z=\overset{m}{\underset{i=1}{\sum }}\lambda_{i}Z_{i}$$

where *Z* is the calculated data of the interpolation points, *m* is the number of sample points taken, *Z_i_* is the data of each sampling point and *λ_i_* is the size of the weight assigned to each sampling point.

#### 2.2.3. Natural Breaks Method

The main standard classification methods are as follows: defined interval, equal interval, quantile, standard deviation method, natural breaks and geometric interval methods. The natural breaks method is based on the univariate classification method in cluster analysis, which minimizes differences within classes whilst maximizing those between classes by calculating the data breakpoints between classes at a certain number of divisions; this method has the advantage of providing the most effective distinction between similar values in the data [26]. Due to the range of output values of the spatial interpolation maps varying in each map, it is not possible to compare air quality conditions under the same criteria. Therefore, the natural breaks method was chosen to reclassify the image ranges into 40 levels, based on the median values of all interpolated ranges for each map from 2016 to 2020, with the aim of highlighting regional differences as visually as possible. The color scheme of each interpolated map was then adjusted with the new grading criteria so that they could be grouped into one map with the same legend criteria.

## 3. Results

### 3.1. Lag Effect Analysis

**Figure 2:**
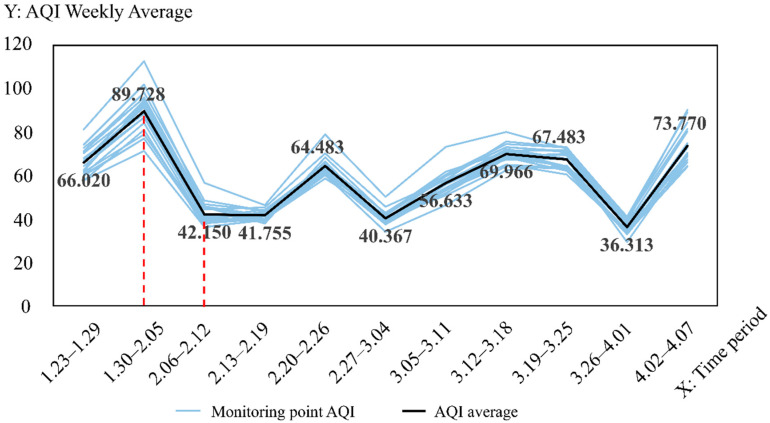
Wuhan AQI weekly change folding chart during the city closure in 2020.

As shown in Figure 2, the weekly average value of AQI in 2020 for 21 monitoring stations in Wuhan City is counted, and its change is represented by the grey broken line; the average value of AQI for all monitoring stations in the same week represents the air quality level in Wuhan City in that week, and its weekly change is represented by the black broken line, which demonstrates the following. (1) Although differences were found between the high and low AQI weekly averages at each point, the overall trend is consistent, and using the average value to represent the air quality level in Wuhan is meaningful and representative. (2) The highest level of overall AQI was from 30 January to 5 February, whilst the weekly average AQI value significantly decreased by the next study period (6–12 February). Meanwhile, the two aforementioned periods demonstrated the largest difference between any two adjacent weeks, presumably with the influence of a possible lag effect in this period.

**Figure 3:**
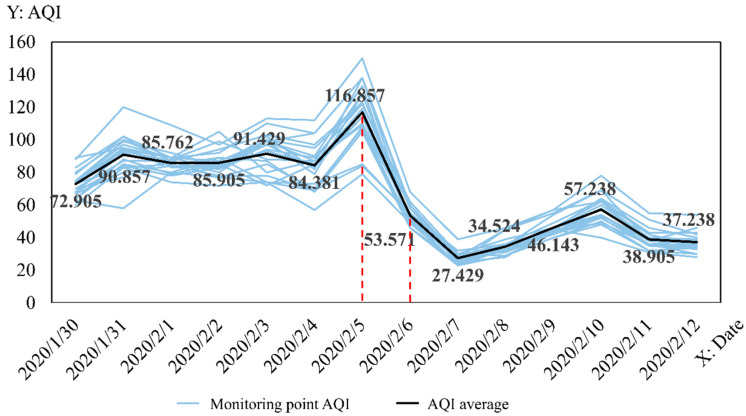
Wuhan City, 30 January 2020–12 February 2020, AQI daily change folding line graph.

The daily AQI values of each monitoring point and the daily average AQI values of all monitoring points in this period are represented as shown in Figure 3 to observe in detail the AQI changes in Wuhan from 30 January to 12 February 2020. In this figure, the grey broken line indicates the daily AQI changes in each monitoring point, and the black broken line indicates the daily changes in the average AQI values of all monitoring points. The figure reveals that the AQI values of each point generally reached the maximum on 5 February and rapidly decreased on 6 February; the trend of the broken line of each point was flatter and closer compared with the previous trend from 6 February. The statistical data revealed that the average AQI range before 5th and after 6th were respectively 72.905–116.857 and 27.429–57.238. The data difference is observed. Therefore, the change point of the lag effect is speculated to 5 February.

**Figure 4:**
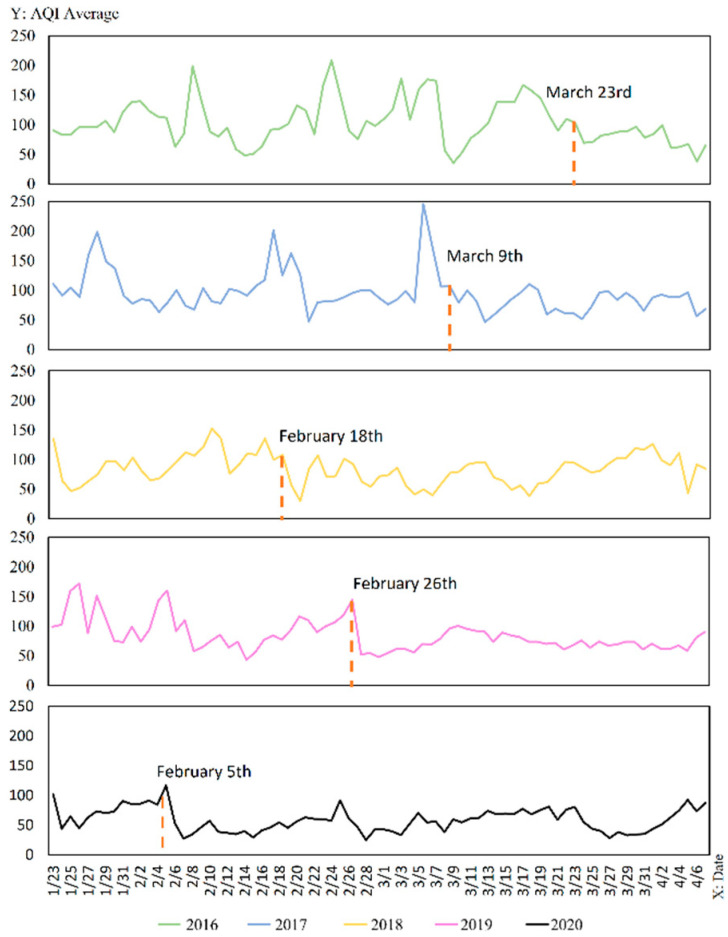
Wuhan City 2016–2019 vs. 2020 AQI change during city closure year-on-year folding chart.

AQI changes can be influenced by seasonal factors [18,19]. The AQI for the same periods from 2016 to 2020 were shown in Figure 4 to rule out that the sudden changes in AQI values are not due to seasonal factors. The figure revealed that not significantly similar trend in AQI values existed from 2016 to 2020. The mutation points for each year were derived from the Pettitt mutation test, and the results are labelled in the Figure 4, with different AQI mutation points from 23 January to 7 April for each year from 2016 to 2020. Overall, the sudden change of AQI in 2020 is not caused by seasonal factors but is attributed to the lag effect, and its change point is on the 14th day after the city closure (i.e., 5 February 2020).

### 3.2. Spatial and Temporal Divergences of AQI

**Figure 5:**
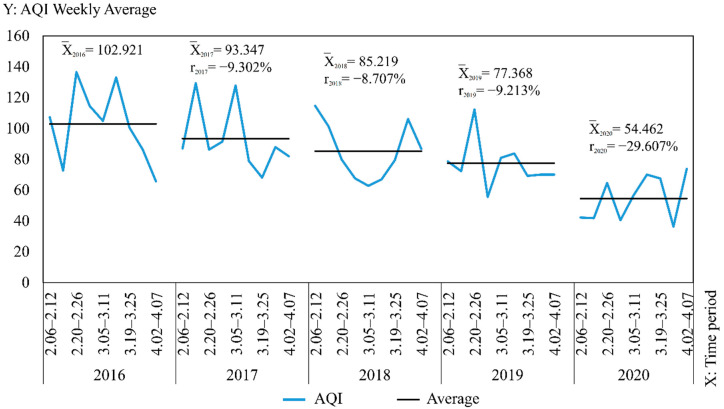
AQI weekly average year-on-year change folding chart from 2016 to 2020.

Excluding the 14 days with lag, the study time range was adjusted to 6 February–7 April according to the results in Section 3.1, and the daily AQI level in Wuhan was expressed as the average AQI values from 21 monitoring stations per day. The average AQI values ($\bar{x}$) and growth rates (r) were counted in seven days to generate a line graph of the year-on-year change in weekly AQI averages from 2016 to 2020, as shown in Figure 5. In this figure, the averages are marked on the graph in the form of black straight lines and notes, indicating the specific changes in AQI levels for each week. The growth rates are the rates of increase compared to the same period of the previous year and are marked on the graph in the form of notes. The results show that the average AQI values show a trend of year-by-year decrease during the study period, and the growth rates in each year from 2016 to 2019 are relatively similar at −9.302%, −8.707%, and −9.213%. However, the decrease in 2020 is larger relative to the previous year, with the growth rate of −29.607%. This finding indicates that although a year-by-year improvement in air quality is observed, the reduction in human activity caused by the city closure in 2020 induced a significant drop in the AQI values, and the air quality condition during the city closure is significantly better than in other years.

Counting the number of days with AQI index categories of excellent and good and calculating the excellent rate from 6 February to 7 April for each year from 2016 to 2020 as shown in Table 2. The average value of the 21 monitoring stations is used to represent the air quality condition of Wuhan for a day. The excellent rate is calculated by taking the number of days in the statistical period (6 February–7 April) as the denominator and the number of days with AQI values less than or equal to 100 (i.e., the index categories of excellent and good) as the numerator. The results show that the excellent air quality rate increased yearly from 2016 to 2020, and the number of days with excellent air quality was higher in 2020 compared to other years. This finding shows that the air quality condition during the city closure in 2020 was better than the same period in other year, and the city closure promotes the improvement of air quality condition.

**Figure 6:**
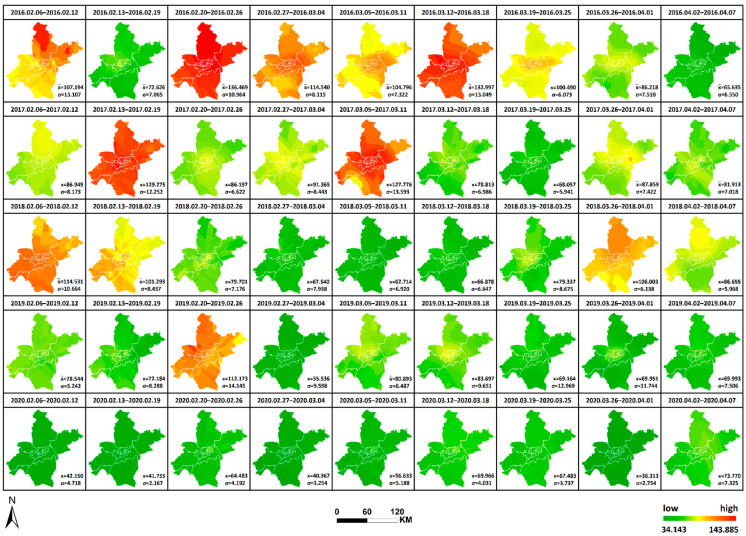
AQI interpolation chart from 2016 to 2020.

Similarly, the daily AQI levels in Wuhan were expressed as the average of AQI from 21 monitoring stations per day, and the ordinary Kriging interpolation method was used to generate 9 × 5 AQI interpolation maps by week for AQI data from 6 February to 7 April of each year from 2016 to 2020 in Wuhan (as shown in Figure 6). The horizontal changes in AQI each year and the vertical comparison of AQI yearly in the same period can be observed, facilitating a comprehensive analysis of the spatial and temporal variation characteristics of AQI in Wuhan from 2016 to 2020 and the differences in air quality conditions in time and space during the city closure in 2020 compared with other years. The interpolation results are re-graded using the natural breaks method. Therefore, all interpolation maps are generated under a uniform and reasonable legend standard, where AQI = 100 is used as the divide in yellow, AQI < 100 is denoted by green (a low value is indicated by dark green) and AQI > 100 is denoted by red (dark red indicates a high value). The mean value ($\bar{x}$) of AQI and the standard deviation (σ) of the weekly mean value at each station relative to the mean value $\bar{x}$ were calculated and added to Figure 6, thus reflecting the degree of data dispersion relative to the mean and allowing a quantitative analysis of air quality equilibrium in 2020 compared with other years during the selected study period. The maximum or minimum values, the corresponding time and region and the range and annual standard deviation were counted as shown in Table 3, facilitating the observation of geographical distribution and variation of the high and low values. The following results were obtained: (1) Compared with other years, the standard deviation in 2020 is the lowest in all weeks except the final week, and the overall standard deviation in 2020 is also significantly lower than that in other years. This phenomenon indicates that the air quality condition is superior during the city closure, whilst the geographical differences are also markedly reduced and balanced, and the previously heavily polluted areas are significantly reduced by the city closure. (2) Unlike other years when the highest value occurs in February, the highest value in 2020 occurs in April. In addition, the two weeks with extreme values in 2019 and 2020 are adjacent to each other. In 2019, the highest values occur first, followed by the lowest value. On the contrary, in 2020, the lowest values occur first, followed by the highest values. The maximum occurs before unblocking, reflecting the gradual return of human activities.

### 3.3. Spatial and Temporal Divergences of Pollutants

**Figure 7:**
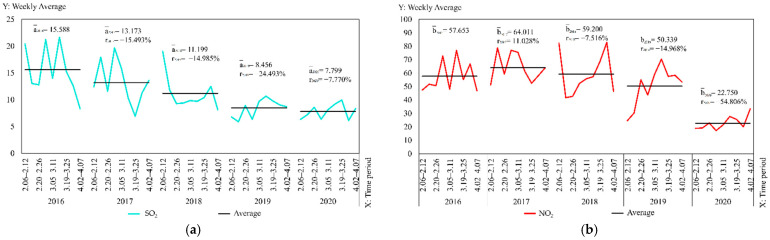

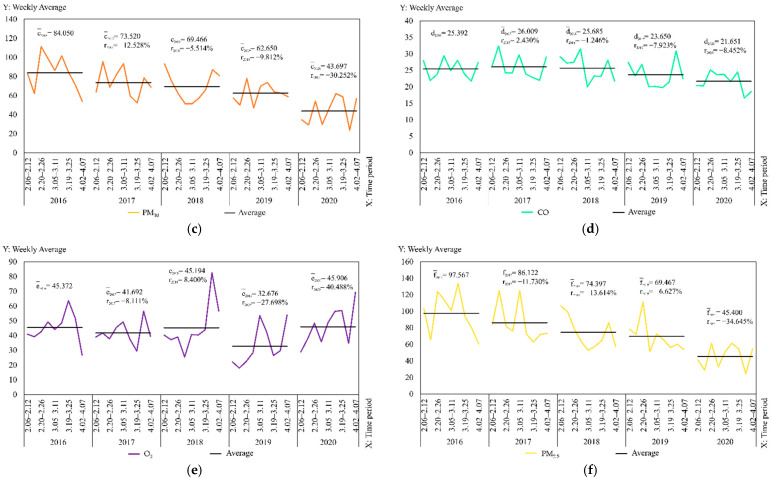
Weekly pollutant average year-on-year change folding chart from 2016 to 2020: (**a**) SO_2_; (**b**) NO_2_; (**c**) PM_10_; (**d**) CO; (**e**) O_3_; (**f**) PM_2.5_.

The average values and growth rates of the six pollutants from 6 February to 7 April of each year from 2016 to 2020 were counted separately to further understand the temporal changes of air pollutants in Wuhan. The values are displayed visually in the form of a line graph as shown in Figure 7. The following results were obtained: (1) the decrease in NO_2_, PM_10_ and PM_2.5_ is significantly larger in 2020, and the main sources of these pollutants are dust emissions from vehicle exhaust or driving, industrial emissions and fuel combustion [27,28,29]. The significant decrease is attributable to the reduction of major sources of these pollutants due to the city closure. (2) SO_2_ and CO are produced in the process of fuel combustion [30,31], but their changes are insignificant since their levels in the air are lower compared with those of other pollutants. (3) VOCs and NOx are key precursors for O_3_ production [32]. O_3_ and other secondary pollutants are produced by chemical reactions under the action of light [33]. The main sources of O_3_ include vehicle exhaust, oil and gas volatilization and industrial processes. Meanwhile, factors such as meteorological conditions and weather systems, affect O_3_ levels to some extent [32]. The data in this paper show an increase in O_3_ values in 2020 compared with those in the previous four years, which is similar to the findings of Cao and Feng et al. on the effect of pandemic prevention and control measures on O_3_ changes. Sicard et al. showed that the increase in urban O_3_ concentrations can be attributed to the reduction in regional nitrogen oxide (NOx) emissions [34]. The study by Witte et al. similarly concluded that O_3_ increases with increasing VOCs and decreases with increasing NOx [35]. NO_2_ values dropped considerably during the blockade, so the reason for increasing O_3_ could be influenced by decreasing NOx, which is an issue that should still be studied comprehensively.

**Figure 8:**
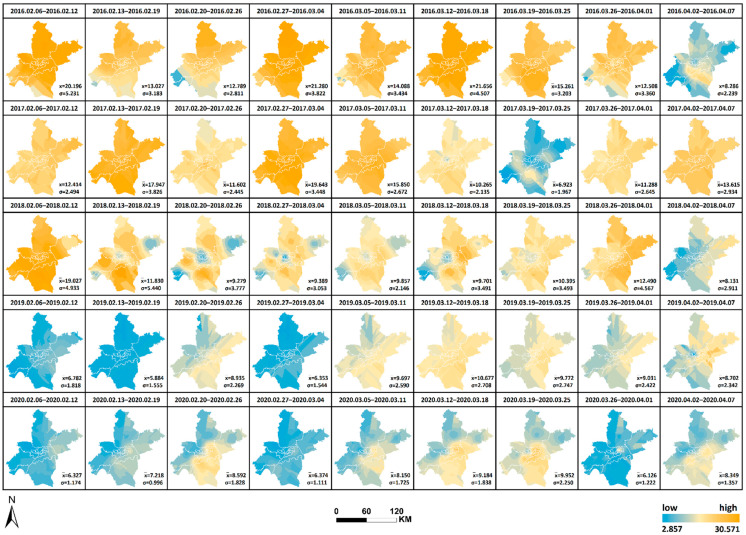
SO_2_ interpolation chart from 2016 to 2020.

**Figure 9:**
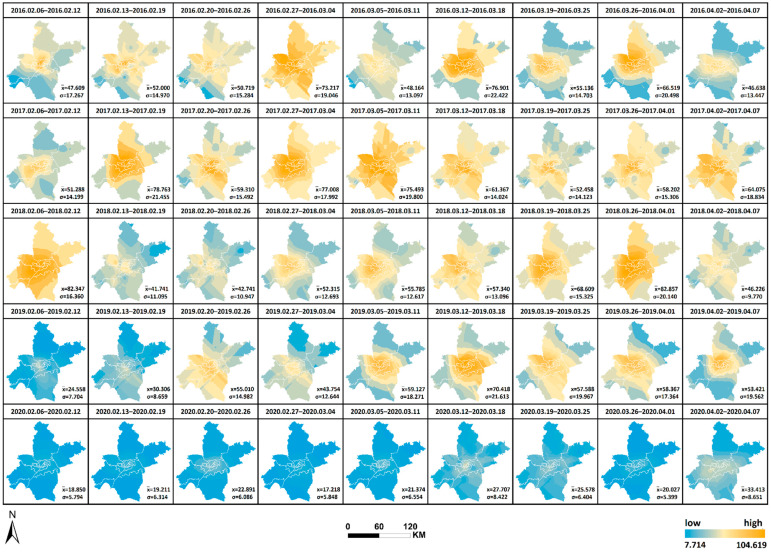
NO_2_ interpolation chart from 2016 to 2020.

**Figure 10:**
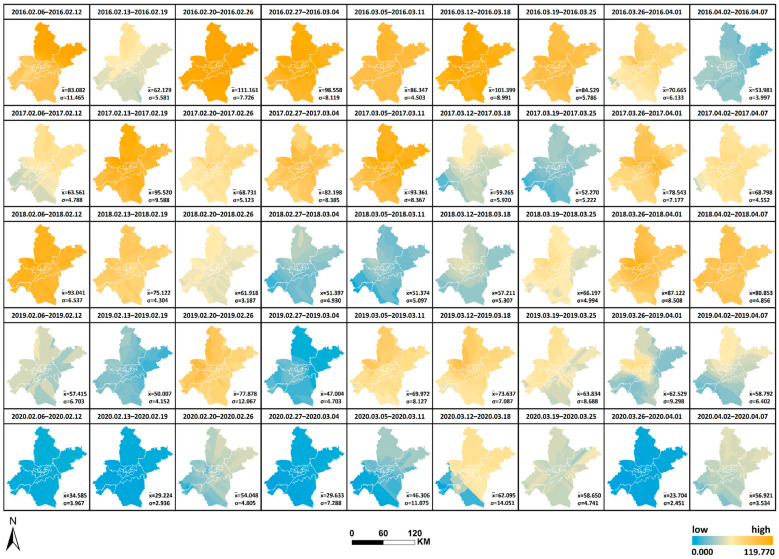
PM_10_ interpolation chart from 2016 to 2020.

**Figure 11:**
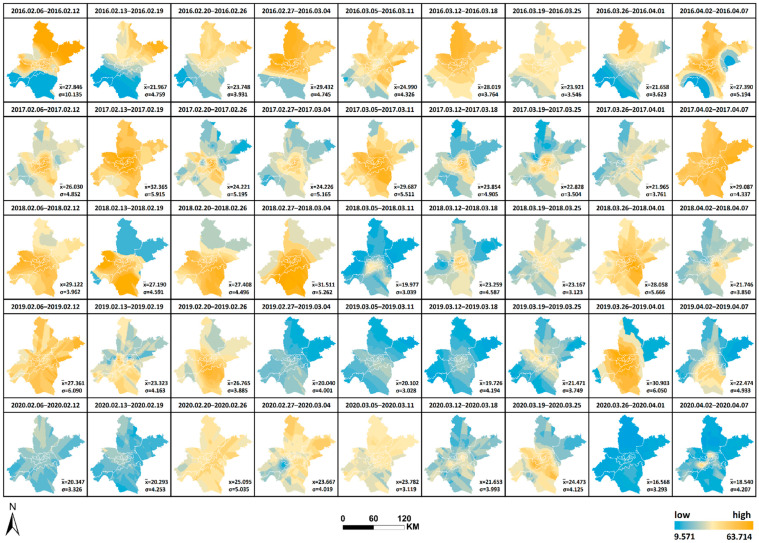
CO interpolation chart from 2016 to 2020.

**Figure 12:**
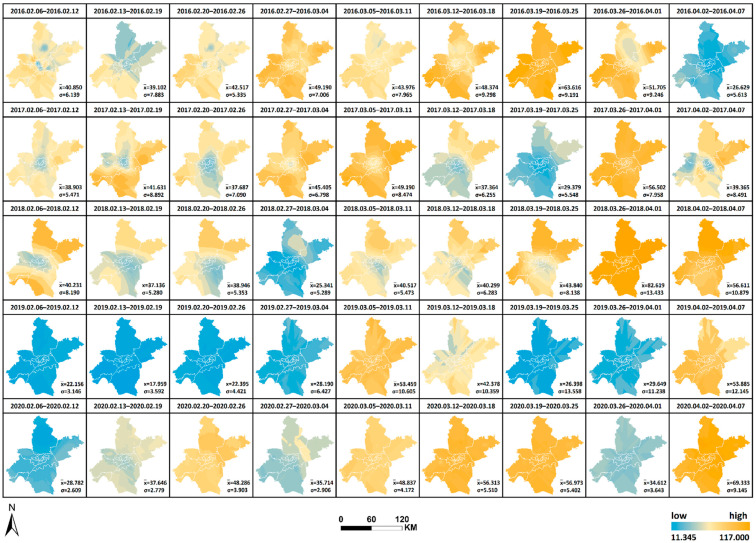
O_3_ interpolation chart from 2016 to 2020.

**Figure 13:**
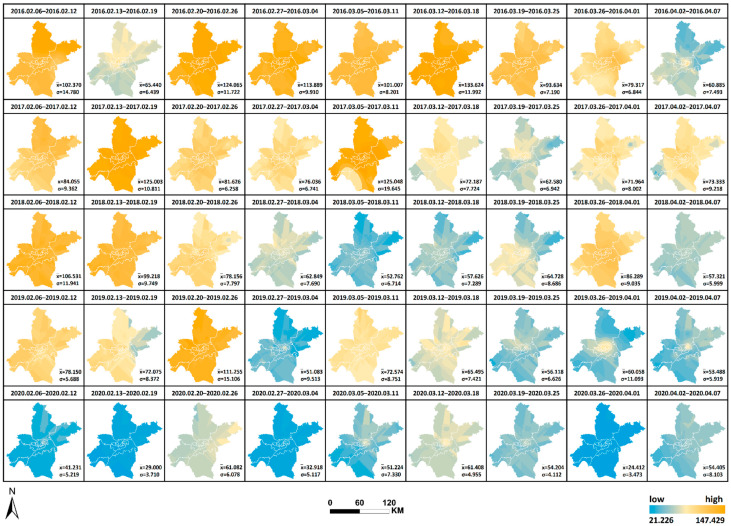
PM_2.5_ matter interpolation chart from 2016 to 2020.

The spatial and temporal changes and comparative differences of each pollutant from 6 February to 7 April yearly from 2016 to 2020 are comprehensively analyzed. The interpolation maps of six pollutants were generated by week through ordinary Kriging interpolation with the pollutant monitoring values of 21 monitoring stations as the base data, and the interpolation results were re-graded by the natural breaks method. The changes in values from low to high were indicated by two colors of blue and orange, as shown in Figure 8, Figure 9, Figure 10, Figure 11, Figure 12 and Figure 13, which visually demonstrate the temporal and spatial variations of various pollutants in different regions of Wuhan city. The mean values ($\bar{x}$) of each pollutant and the standard deviation (σ) relative to mean values $\bar{x}$ at the 21 stations were calculated and summarized, which were added to the interpolation maps. The standard deviation allows the quantification of the spatial equilibrium of each pollutant in 2020 compared with other years in the selected study period. The maximum/minimum of each pollutant, the corresponding time and region and the range and annual standard deviation are counted as shown in Table 4, Table 5, Table 6, Table 7, Table 8 and Table 9, facilitating the geographic observation of the distribution and variation of high and low values.

The following results are also obtained. (1) The standard deviation of SO_2_ in Wuhan during the city closure in 2020 is the lowest in all week, except for the seventh week, whilst the overall standard deviation is lower than that of other years. The standard deviation of NO_2_ in all week and the overall deviation are substantially lower than that of other years. The standard deviation of CO is the lowest in only two weeks, but the overall deviation is lower than that of other years. The standard deviation of O_3_ is the lowest in all week, except for the ninth week, whilst the overall standard deviation is lower than that of other years. The standard deviation of PM_2.5_ is the lowest in all week, except for the fifth and ninth weeks, and the overall standard deviation is substantially lower than that of other years. These findings indicate that the geographical differences of these pollutants decrease and the tendency is equalized in 2020, and the change is evident in the areas with severe pollution in this category. Meanwhile, the change of PM_10_ does not show obvious variations of geographical differences. (2) The maximum and minimum of O_3_ in 2020 is at the high level in five years. The maximum of all pollutants in 2020, except O_3_, is at the lowest in five years. The minimum of all pollutants in 2020, except O_3_ and SO_2_, is at the lowest in five years, wherein the minimum of inhalable particles is down to 0. (3) Contrary to 2016–2019, the lowest and highest values of O_3_ pollution in 2020 are, respectively, located in the distant and central urban areas; contrary to 2016–2019, the lowest value of fine particle pollution in 2020 is in the central urban areas.

Figure 8, Figure 9, Figure 10, Figure 11, Figure 12 and Figure 13 Pollutants interpolation chart from 2016 to 2020: SO_2_; NO_2_; PM_10_; CO; O_3_; PM_2.5_.

Table 4, Table 5, Table 6, Table 7, Table 8 and Table 9 Pollutants maximum/minimum statistics from 2016 to 2020: SO_2_; NO_2_; PM_10_; CO; O_3_; PM_2.5_.

## 4. Discussion

We compared the air quality data for the five years from 2016 to 2020 and found that overall air quality conditions in Wuhan substantially improved impacted by city closure. This study could be further discussed in the following areas.

We started this study at the beginning of 2021, so the deadline for collecting data was the end of 2020. Theoretically, the richer the data from different years, the more comparative value it has. In China, every five years, the government will make a development plan, and it is reasonable to take a five-year cycle to observe the development of a city in China and its resulting changes in air quality. Therefore, we chose to collect data for the period 2016–2020 for our study. Missing air quality monitoring data is inevitable. Various methods have been proposed to fill in these missing data, such as mean filling, the regression analysis method, EM filling algorithm, multiple interpolation, KNN-DBSCAN algorithm, NN-DSAE algorithm, GRU, etc. [36,37,38,39]. The arithmetical series formula is relatively simple and commonly used methods. The missing data in this paper are less than 3% of the total data, and it remains to be verified whether the use of different methods will have an influence on the results of the study.

Xu et al. tested the lagged correlation of AQI on confirmed COVID-19 cases by lagged models [40]. Ma et al. applied the distributed lag nonlinear model and generalized summation model to calculate the lag effect of AQI on the number of respiratory emergencies [41]. Bao et al. used a Poisson regression model combined with the distributed lag nonlinear model to study the lag effect of air pollution on population mortality [42]. However, studies analyzing the lag effect of urban blockade on AQI are relatively few. There are various methods for lag effects analysis, such as DLNM and lagged variable model [43,44]. In this study, the mutation test was used. At present, the Pettitt mutation test has been applied very little to air quality. Shi made the mutation point analysis of PM2.5 concentration time series with the help of MK mutation test and Pettitt mutation test [45]. Luo et al. suggested that the MK mutation test could excellently reveal the mutation characteristics of AQI [46]. This study used four methods, including MK Mutation Test, Pettitt Mutation Test, Buishand U Test, and Standard Normal Homogeneity Test (SNHT). The latter three methods mentioned above generated generally consistent results, but both the Buishand U Test and SNHT had one result that differed from the others. Meanwhile, with reference to the trend of AQI reflected in the graph of MK Mutation Test, the results of the Pettitt mutation test were used in this study.

This study and most related studies in other cities have similar findings. Same as the study by Cao, Feng and Lian et al. [14,15,47], this study found an increase in O_3_ values in Wuhan during the city closure and the main reason for this phenomenon may be related to the change in NOx, which is yet to be further investigated. However, this study did not take into account other factors that influence AQI, including temperature, wind, regional transport patterns and precipitation that are not identical for each year among years.

Considering the following aspects is reasonable. (1) The method used to deal with the residual data is arithmetical series formula. However, these type of data are rare, which may also have an impact on the study results. (2) The main reasons for the increase in ozone values must be comprehensively studied. (3) Further studies regarding the correlation between air quality and different human activities (whether a pattern in the spatial and temporal variation of AQI exists after the unlocking of cities and the possibility of assessing the recovery of people’s production life) can be conducted.

## 5. Conclusions

Unlike existing studies, this paper considers not only seasonal shifts but also lag effects when analyzing the effects of city closure measures on urban air quality. The following main findings are presented.

1. The change point for the lagging effect of the urban closure in Wuhan is on the 14th day after the closure (5 February 2020);
2. Air quality conditions during the city closure in 2020 were significantly better than that in other years, indicating that the reduction of human activities due to the city closure had a positive effect on the improvement of air quality conditions;
3. The overall air quality conditions are more balanced in 2020 compared with those in 2016–2019, the geographical differences are reduced, the air quality improvement is evident in the previously heavily polluted areas affected by the urban blockade and the highest AQI value in 2020 occurred before the unblocking, reflecting the gradual return of human activities;
4. The decrease in NO_2_, PM_10_, and PM_2.5_ during the city closure in 2020 is significantly large, the change in SO_2_ and CO is not observed and the O_3_ value is the highest compared with other years;
5. The following were observed during the closure of the city in 2020: the geographical differences of SO_2_, NO_2_, CO, O_3_, and PM_2.5_ decreased and tended to be balanced, the changes in the areas with serious pollution of this type were evident, the changes in PM_10_ did not show obvious variations in geographical differences and the extreme value of O_3_ pollution and the minimum of PM_2.5_ pollution in 2020 showed the opposite distribution pattern with other years.

## Figures and Tables

**Table 1:** Summary of data collection numbers.

	2016	2017	2018	2019	2020
Number of data available for collection	1575	1525	1563	1424	1595
Number of missing data	21	50	12	151	1

**Table 2:** Overall air quality excellent rate statistics from 2016 to 2020.

	2016	2017	2018	2019	2020
Number of days with excellent	3	2	7	2	26
Number of days with good	33	41	37	52	36
Excellent rate	58.065%	70.492%	72.131%	88.525%	100.000%

**Table 3:** AQI maximum/minimum statistics from 2016 to 2020.

		2016	2017	2018	2019	2020
maximum	value	155.143	147.857	131.429	139.500	90.333
	time	2.20–2.26	2.13–2.19	2.06–2.12	2.20–2.26	4.02–4.07
	region	Qingshan	Caidian	Wuchang	Qiaokou	Hongshan
minimum	value	53.167	56.857	51.714	40.167	29.429
	time	4.02–4.07	3.19–3.25	3.05–3.11	2.27–3.04	3.26–4.01
	region	Xinzhou	Caidian	Hongshan	Huangpi	Jianghan
range		101.976	91.000	79.714	99.333	60.905
annual standard deviation		43.252	35.977	28.653	23.725	17.914

**Table 4:** SO_2_ maximum/minimum statistics from 2016 to 2020.

		2016	2017	2018	2019	2020
maximum	value	30.571	26.714	31.000	17.909	15.143
	time	3.12–3.18	2.13–2.19	2.13–2.19	3.19–3.25	3.19–3.25
	region	Wuchang	Hongshan	Jiangxia	Hongshan	Qingshan
minimum	value	4.500	3.286	3.167	2.583	4.000
	time	4.02–4.07	3.19–3.25	4.02–4.07	2.27–3.04	3.26–4.01
	region	Caidian	Caidian	Jianghan	Jianghan	Wuchang
range		26.071	23.429	27.833	15.326	11.143
annual standard deviation		8.344	6.828	6.848	3.437	2.812

**Table 5:** NO_2_ maximum/minimum statistics from 2016 to 2020.

		2016	2017	2018	2019	2020
maximum	value	103.429	104.143	110.143	101.909	48.333
	time	3.12–3.18	2.13–2.19	3.26–4.01	3.19–3.25	4.02–4.07
	region	Qiaokou	Caidian	Qiaokou	Jianghan	Caidian
minimum	value	15.571	21.000	17.571	10.143	7.714
	time	2.20–2.26	2.20–2.26	2.13–2.19	2.06–2.12	2.27–3.04
	region	Caidian	Caidian	Xinzhou	Huangpi	Caidian
range		87.857	83.143	92.571	91.766	40.619
annual standard deviation		28.670	26.275	28.887	24.552	10.310

**Table 6:** PM_10_ maximum/minimum statistics from 2016 to 2020.

		2016	2017	2018	2019	2020
maximum	value	122.714	115.714	108.286	99.000	69.714
	time	2.20–2.26	2.13–2.19	2.06–2.12	2.20–2.26	3.12–3.18
	region	Jianghan	Jianghan	Jianghan	Dongxihu	Dongxihu
minimum	value	45.000	39.857	33.857	36.000	0.000
	time	4.02–4.07	3.19–3.25	3.05–3.11	2.27–3.04	2.27–3.18
	region	Xinzhou	Caidian	Caidian	Hongshan	Caidian
range		77.714	75.857	74.429	63.000	69.714
annual standard deviation		33.325	23.613	23.607	17.161	18.231

**Table 7:** CO maximum/minimum statistics from 2016 to 2020.

		2016	2017	2018	2019	2020
maximum	value	63.714	43.571	46.714	41.286	38.857
	time	2.06–2.12	2.13–2.19	3.26–4.01	2.06–2.12	2.20–2.26
	region	Xinzhou	Qingshan	Hongshan	Qiaokou	Hangyang
minimum	value	11.571	13.714	12.714	13.000	9.571
	time	2.06–2.12	3.26–4.01	3.05–3.11	2.27–3.04 & 3.19–3.25	3.26–4.01
	region	Jiangxia	Hangyang	Caidian	Jianghan & Xinzhou	Huangpi
range		52.143	29.857	34.000	28.286	29.286
annual standard deviation		9.646	7.981	8.185	7.457	6.631

**Table 8:** O_3_ maximum/minimum statistics from 2016 to 2020.

		2016	2017	2018	2019	2020
maximum	value	81.429	66.714	117.000	71.714	89.500
	time	3.19–3.25	3.26–4.01	3.26–4.01	3.05–3.11	4.02–4.07
	region	Xinzhou	Jiangxia	Huangpi	Caidian	Hongshan
minimum	value	14.857	20.000	18.667	4.000	19.429
	time	2.13–2.19	3.19–3.25	2.27–3.04	3.19–3.25	2.06–2.12
	region	Jiang’an	Jianghan	Jianghan	Jianghan	Huangpi
range		66.571	46.714	98.333	67.714	70.071
annual standard deviation		20.855	18.646	23.438	21.769	18.404

**Table 9:** PM_2.5_ maximum/minimum statistics from 2016 to 2020.

		2016	2017	2018	2019	2020
maximum	value	151.333	147.429	127.571	139.500	79.000
	time	2.20–2.26	3.05–3.11	2.06–2.12	2.20–2.26	2.20–2.26
	region	Qingshan	Qingshan	Qingshan	Qiaokou	Qingshan
minimum	value	46.667	42.333	41.857	37.167	19.714
	time	4.02–4.07	4.02–4.07	3.05–3.11	2.27–3.04	3.26–4.01
	region	Xinzhou	Caidian	Xinzhou	Huangpi	Hangyang
range		104.667	105.095	85.714	102.333	59.286
annual standard deviation		47.246	39.284	30.239	25.540	17.922

## Data Availability

The air quality data used in this paper were obtained from the official website of the Wuhan Ecological Environment Bureau (http://hbj.wuhan.gov.cn/, accessed on 1 February 2021).
